# Clear cell myomelanocytic tumor of ligamentum teres

**DOI:** 10.4322/acr.2024.503

**Published:** 2024-06-21

**Authors:** Ariba Zaidi, Debajyoti Chatterjee, Venu Bhargav, Vikas Gupta, Ashim Das

**Affiliations:** 1 Dr Ram Manohar Lohia Institute of Medical Sciences, Department of Pathology, Lucknow, Uttar Pradesh, India; 2 Post Graduate Institute of Medical Education and Research, Department of Histopathology, Chandigarh, India; 3 Post Graduate Institute of Medical Education and Research, Department of General Surgery, Chandigarh, India

**Keywords:** Immunohistochemistry, Liver Neoplasms, Perivascular Epithelioid Cell Neoplasms

## Abstract

Clear cell myomelanocytic tumor (CCMMT) of the falciform ligament/ligamentum teres is a rare hepatic tumor, a variant of the perivascular epithelioid cell tumor (PEComa) family. CCMMT is the rarest variant of hepatic PEComas. Only a few cases of CCMMT have been reported in the English literature. Because of its rarity, less is known about its biological behavior. We present a case of a 31-year-old female who complained of abdominal pain, bilious vomiting, and abdominal fullness over two months. The radiological impression was of focal nodular hyperplasia. The histological examination of the resection specimen revealed a well-circumscribed tumor arranged in fascicles, sheets, and a whorling pattern. The tumor cells were spindle to epithelioid shaped with abundant clear to pale eosinophilic cytoplasm. The tumor cells expressed both myoid (smooth muscle actin) and melanocytic (MelanA and HMB45) markers, while they were negative for hepatocytic and vascular markers. Thus, based on histology and immunohistochemistry, a diagnosis of CCMMT was made. This case presents the diagnostic challenges of CCMMT and discusses the differential diagnosis with a literature review.

## INTRODUCTION

Clear cell myomelanocytic tumor (CCMMT) of the falciform ligament/ligamentum teres is a rare liver tumor and is a variant of perivascular epithelioid cell tumor (PEComa). CCMMT was first described in the year 2000. Hepatic PEComas are rare tumors, and CCMMT is the rarest variant of hepatic PEComas. These tumor cells have spindle to epithelioid appearance with clear to pale eosinophilic cytoplasm and characteristically express both myoid (smooth muscle actin) and melanocytic (MelanA and HMB45) markers. Only a very few cases of CCMMT have been reported in the English literature. Not much is known about the biological behavior of this rare tumor, as only a few case reports are available in the literature. Here, report a case of CCMMT in a young female with a histological differential diagnosis and a literature review. We aim to study this tumor’s morphological features and outcome in the light of existing literature.

## CASE REPORT

**Figure 1:**
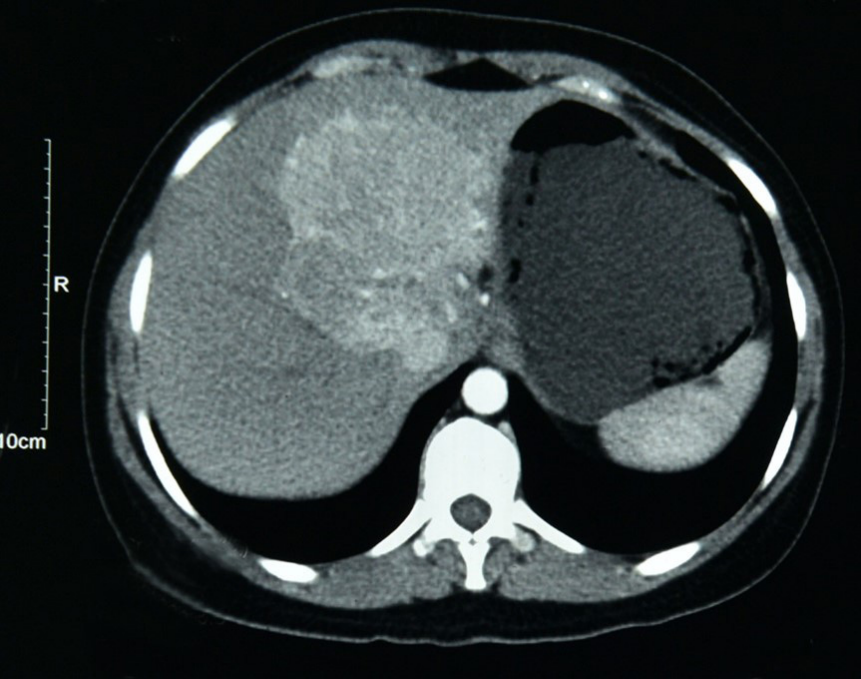
Abdominal computed tomography scan showing a large, lobulated tumor involving the left lobe and part of the right lobe of the liver.

A 31-year-old female presented with abdominal pain, bilious vomiting and abdominal fullness over two months. No associated co-morbidities were present. She denied intake of oral contraceptives or steroids. Per abdominal examination, the liver was palpable 2 cm below the right costal margin. The abdominal ultrasonography (USG) showed an enlarged liver, with a liver span measuring 15.9 cm. An ill-defined hyperechoic lesion of 5.5 x 6 cm with few calcific foci was seen in the right liver lobe. On performing an abdominal contrasted enhancing computed tomography (CECT), a large lobulated mass lesion measuring 6.9 x 7.3 x 6.7cm was identified involving the ligamentum teres and segments II, VII, IVa, caudate lobe of the liver, and (Figure 1).

It showed arterial hypervascularity with a central isodense area on the portal vein and delayed phase images with a delayed enhancement of the lesion periphery. The lesion caused splaying of the middle and left hepatic veins with compression of the left branch of the portal vein. There was no intrahepatic biliary radical dilatation. The clinical and radiological impression of focal nodular hyperplasia (FNH) versus hepatic adenoma. Serum alpha-fetoprotein (AFP) was normal, and serology for hepatitis B and C was negative. This patient had no clinical features or family history of Tuberous Sclerosis. Fundus and dermatological examinations did not reveal any features suggesting Tuberous Sclerosis coexistence. However, genetic testing of TSC1 or TSC2 genes mutation was not done.

Modified extended left hepatectomy was done under general anesthesia, and the specimen was sent for histopathological examination.

**Figure 2:**
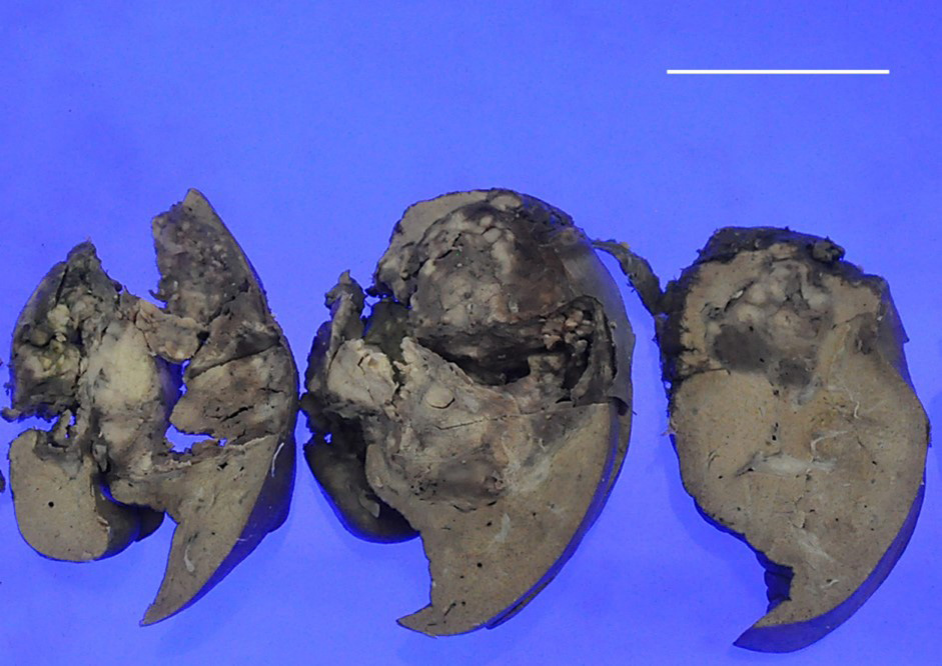
Gross image of the slices of the liver showing a large mass in the right lobe reaching up to the liver capsule with central hemorrhagic areas. Scale bar= 10cm.

The specimen measured 13 x 10 x 8 cm on gross examination. A relatively circumscribed lesion, measuring 10 x 8 x 7cm, was identified in the region of ligamentum teres and involving the liver (Figure 2). Most of the portion of ligamentum teres was absorbed into the mass. It was grey-white to brown with areas of hemorrhage, soft to firm in consistency, and friable in places. This mass reached up to the capsule of the liver. The adjacent liver parenchyma was unremarkable.

**Figure 3:**
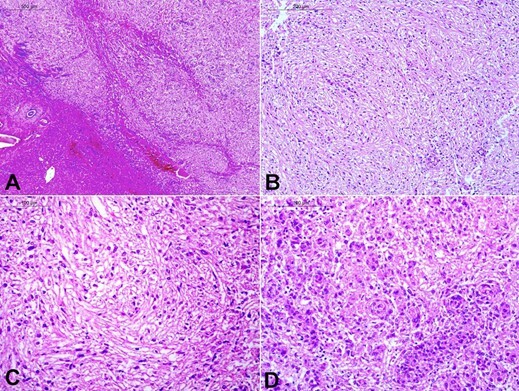
**A –** A well-circumscribed tumor is seen, sharply demarcated from the normal liver (lower left part) (H&E, x40); **B –** The tumor cells are arranged in fascicles (H&E, x100); **C –** Tumor cells are epithelioid in shape and have clear to pale eosinophilic cytoplasm (H&E, x200); **D –** At places, the tumor cells are arranged in a whorling pattern around blood vessels (H&E, x200).

Microscopic examination revealed a well-circumscribed tumor comprising sheets and tumor cells’ fascicles. These tumor cells were spindle-shaped and, at places, showed epithelioid morphology. In some places, the tumor cells showed a whorling pattern, arranged around small blood vessels, and appeared to be arising from those vessels. These tumor cells had clear to eosinophilic cytoplasm, fine chromatin, and conspicuous micronucleoli (Figures 3A-D).

There was no mitosis, necrosis, or significant nuclear pleomorphism. Multiple foci of lymphomononuclear inflammatory infiltrate and thin capillaries throughout the tumor were present. The tumor lacked any portal tracts, unpaired hepatic arteries or normal hepatocytes. Friable areas show hemorrhagic foci, no necrosis was present. The tumor was well circumscribed. No vascular invasion was noted. The adjacent uninvolved liver parenchyma showed normal-appearing hepatocytes and mild portal tract inflammation, with a few portal tracts showing bile ductular proliferation.

**Figure 4:**
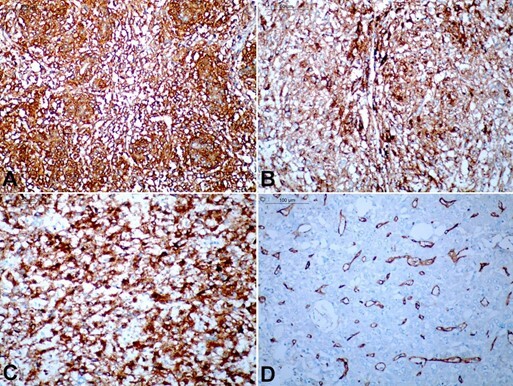
The tumor cells show strong expression of smooth muscle antigen (**A –** immunohistochemistry, x200), HMB-45 (**B –** immunohistochemistry, x400), and MelanA (**C –** immunohistochemistry, x400). (**D**) CD34 stain highlights the capillaries, while the tumor cells are negative (immunohistochemistry, x200).

On immunohistochemistry, these tumor cells were diffuse (>90%) strong positive for HMB45 and patchily (20-30%) strong positive for Melan A. These tumor cells were also positive for smooth muscle actin (SMA), which showed stronger positivity in tumor cells, showing a whorling pattern. The tumor cells were negative for HepPar1, glutamine synthetase, arginase 1, glypican 3, S100, CD34 and CD31. CD34 highlighted the capillaries within the tumor (Figure 4A-D). Based on the histology and immunohistochemistry, a diagnosis of CCMMT of ligamentum teres/falciform ligament was concluded.

## METHODS

We searched articles in PubMed, Scopus, and Google Scholar. The terms used for the search were “Clear cell myomelanocytic tumor of ligamentum teres”, “Clear cell myomelanocytic tumor of ligamentum teres and liver”, “PEComa of ligamentum teres” and “Clear cell myomelanocytic tumor of round ligament of the liver”. We excluded articles that were not in English and articles that reported PEComas on other sites. 12 articles were retrieved and reviewed by two pathologists (AZ and DC) to avoid duplication. 13 cases of Clear-cell myomelanocytic tumor of ligamentum teres were found. Information about age, clinical features and follow up/outcome was extracted and has been summarized in Table 1.

**Table 1:** Cases of clear cell myomelanocytic tumor of liver described in the literature

Ref	Age (y)	Sex	Site	Size (cm)	Follow up (m)	Out come
^1^	6	Female	Right lobe	5	24	Free of disease
^1^	3	Female	Right lobe	5.5	10	Free of disease
^1^	15	Female	Right lobe	8x6x4	6	Free of disease
^1^	21	Female	Right lobe	8.5x6x3.5	24	Free of disease
^1^	10	Female	Right lobe	5x5x5	-	-
^1^	29	Male	Right lobe	20x12x10	12	Lung metastasis
^1^	11	Female	Right lobe	9x5x5	60	Free of disease
^2^	13	Female	Right lobe	9x7x6	22	Free of disease
^3^	29	Female	Right lobe	15.5×9.6×14.2	-	-
^5^	54	Female	Right lobe	8.5x7.5x6.6	36	Free of disease
^10^	36	Female	Right lobe	11	34	Free of disease
^11^	31	Female	Right lobe	1.8	6	Free of disease
^12^	28	Female	Abutting left lobe	10x9x8	60	Free of disease
IC	31	Female	Left and right lobe	10x8x7	6	Free of disease

cm= centimeter; IC= index case; m= months; Ref= reference; y=years.

## DISCUSSION

PEComa is a family of tumors that include angiomyolipoma (AML), clear cell “sugar” tumor of the lung, lymphangioleiomyomatosis, and CCMMT.^1-3^ Out of the various PEComa group of tumors involving the liver, CCMMT is the rarest.^4^ CCMMT of ligamentum teres/falciform ligament is a tumor arising from epithelioid cells surrounding the blood vessels, in relation with these ligaments. Only a few cases of CCMMT of the falciform ligament have been described in the literature. We found 13 cases of hepatic CCMMTs in the English literature, out of which 6 have been reported by Folpe et al.^1^ These cases are briefly described in Table 1. Most often present in young women (mean age: 20.8 years) in the right lobe of the liver as a large mass. Abdominal pain is the most common presenting complaint.^5^

On imaging, these tumors are hypervascular with enhancement during contrasted CT and magnetic resonance imaging (MRI). Contrast-enhanced USG shows early uptake into the tumor with rapid clearance.^6,7^ CCMMTs usually present as a solitary liver tumor. The differential diagnoses include hepatic adenoma, FNH, and hepatocellular carcinoma on imaging.

Histologically, these tumors are characterized by fascicles and nests of spindle-shaped tumor cells having clear to lightly eosinophilic cytoplasm, vesicular chromatin, and prominent micronucleoli. The characteristic histological feature is the epithelioid appearance of tumor cells, which are closely related to dilated vascular channels and expression of myomelanocytic markers. Like to other PEComa tumors family, the cells of CCMMT express melanocytic markers such as HMB-45 and Melan-A and smooth muscle markers such as SMA and h-caldesmon. According to previous studies, HMB-45 is almost always diffusely expressed in these tumors, and Melan A is also expressed in most cases.^1^ On ultrastructural examination, the tumor cells show abundant cytoplasmic glycogen premelanosomes, hemidesmosomes, and poor intracellular junctions.^8,9^ Some tumors of the PEComa group (like angiomyolipoma and lymphangioleiomyomatosis) occur in the background of tuberous sclerosis complex (TSC) syndrome. In addition, many cases of syndromic and sporadic PEComas have loss-of-function mutations in*TSC1*or*TSC2*genes, resulting in activation of the mammalian target of rapamycin (mTOR) pathway, which acts as a target of mTOR inhibitors, which can be used in the treatment of these cases.^13^ Recurrent*RAD51B*gene fusions have been reported only in uterine PEComas.^13^ Infrequently, PEComas can be associated with TFE3 translocations. Such cases particularly lack *TSC* gene mutations. These tumors have a unique morphology and immunophenotype compared to the conventional cases. Tumors with *TFE3* translocation have an alveolar architecture with clear cell morphology and epithelioid tumor cells with round nuclei and clear cytoplasm.^14^ However, the current case only displays a few of these features, like completely clear cell appearance or alveolar architecture. *TFE3* immunostain was not performed in the current case.

The histological differential diagnoses of CCMMT include focal nodular hyperplasia, hepatic adenoma, hepatocellular carcinoma, leiomyoma, and epithelioid hemangioendothelioma. The salient morphological and immunohistochemical features that help distinguish these entities from CCMMT, have been described in Table 2.

**Table 2:** Histological and immunohistochemical features of differential diagnosis of clear cell myomelanocytic tumor

Differential diagnoses	Age/Sex	Gross features	Microscopic features	Immunohistochemical profile
Focal nodular hyperplasia	3^rd^ to 5^th^ decade, females	Usually solitary Multicentricity in 20% case. Subcapsular, grayish-white, depressed scar present	The cellular morphology and relationship between hepatocytes and bile ducts are similar to normal liver. Fibrous septa with thickened vessels divide the lesion into pseudo-lobules. Fibrovascular and ductular areas radiating from the septa.	Arginase and HepPar1 - positive in hepatocytes. Glutamine synthetase- Map like positivity. Alpha1-Antitrypsin positive. Glypican 3, HMB45, MelanA and SMA- negative.
Hepatic adenoma	3^rd^ to 5^th^ decade, females	Usually solitary. Capsulated, different color of lesion as compared to adjacent liver	Well-differentiated hepatocytes with abundant eosinophilic granular cytoplasm. No portal triads or central veins. No connection with the biliary system. Kupffer cells present	Arginase and HepPar1-Positive. Estrogen and Progesterone receptors (75% cases), Androgen receptors (20% cases). Glypican3, HMB45, MelanA and SMA- negative.
Hepatocellular carcinoma	Usually 5^th^ decade, males	Solitary, multinodular, diffuse, and massive forms. Background liver is usually cirrhotic.	Trabecular, solid, or tubular growth. Network of sinusoidal vessels surrounds the tumor cells. Less well differentiated cases show pleomorphism, bizarre mitotic figures, and tumor giant cells. Nucleoli are prominent, and the cytoplasm is scanty and basophilic. Well differentiated forms resemble normal hepatocytes.	AFP, HepPar1, pan-cytokeratin, EMA, Alpha1-antitrypsin, fibrinogen, IgG, transferrin receptor, ferritin, MOC-31, Glypican3, Arginase-Positive. HMB-45, MelanA and SMA -negative.
Leiomyoma	No age or sex predilection	Solitary, well circumscribed, rubbery, and whorled	interlacing fascicles of uniform, spindled cells with a moderate amount of eosinophilic, often vacuolated cytoplasm are evident. Nuclei are plump and elongated with blunt ends (“cigarlike”); mitoses are absent or very rare	SMA, Muscle specific actin- Positive. Glypican3, Arginase, HepPar1, HMB45 and MelanA-Negative.
Epithelioid hemangioendothelioma	Adult, females	Usually multicentric	Neoplastic endothelial cells infiltrate sinusoids and veins, both as tuft-like intravascular proliferations and as fibro thrombotic occlusions. These cells are plump, with an acidophilic cytoplasm that is often vacuolated	Endothelial markers such as podoplanin (D2-40), CD31, CD34- Positive. SMA- occasionally positive.
				Glypican3, Arginase, HepPar 1, HMB45, MelanA- Negative
Clear cell myomelanocytic tumor	Young (3^rd^ decade), females	Solitary, large masses, usually in the right lobe	Fascicles and nests of spindle shaped tumor cells with having clear to lightly eosinophilic cytoplasm, vesicular chromatin and prominent micronucleoli. Epithelioid appearance of tumor cells which are closely related to dilated vascular channels	HMB45, Melan A, SMA, H-Caldesmon-Positive.
				Glypican3, arginase and HepPar 1-Negative.

AFP- Alpha-Fetoprotein; EMA-epithelial membrane antigen; Hep Par1- Hepatocyte Paraffin 1; HMB45- Human Melanoma Black-45; SMA- Smooth muscle actin.

CCMMTs are so rare that their biological behavior is not fully understood. However, most of the tumors described in the literature have behaved favorably, while one case showed lung metastasis (Table 1). According to Folpe et al.,^1^ large tumor size, marked pleomorphism, hypercellularity, nuclear atypia, high mitosis, atypical mitosis, coagulative necrosis, infiltrative growth pattern, and large tumor could favor a malignant potential. The present case did not show most of these malignant features except the large size, i.e., 10 x 8 x7 cm, which may be considered a feature of the risk group of "Unknown malignant potential." However, the patient is currently doing well without any clinical or radiological evidence of recurrence or metastasis at the 6-month follow-up. Longer follow-up is required due to the risk of "unknown malignant potential" in such cases.

## CONCLUSION

CCMMT of falciform ligament/ligament teres is a rare hepatic tumor belonging to PEComa family. Morphologically, diagnosis requires a high degree of suspicion, supplemented by immunohistochemical markers. Due to its rarity, the biological behavior remains uncertain, and the treatment and follow-up protocol remains challenging. Hence, these tumors should be considered tumors with uncertain malignant potential and should have a long-term follow-up.
